# Building blocks for better biorepositories in Africa

**DOI:** 10.1186/s13073-023-01235-x

**Published:** 2023-11-06

**Authors:** Talishiea Croxton, Emmanuel Jonathan, Kareemah Suleiman, Olasinbo Balogun, Petronilla J. Ozumba, Sharley M. Aloyo, Gideon Nsubuga, Rogers E. Kamulegeya, Lwanga Newton, John Mukisa, Mukthar Kader, Vuyo Damaneite, Sunji Nadoma, Ezenwa James Onyemata, Abbas Abel Anzaku, Emmanuel Nasinghe, Jennifer Troyer, Bonnie R. Joubert, Christine Beiswanger, Moses L. Joloba, Elizabeth Mayne, Alash’le Abimiku, H3Africa Biorepository PI Working Group

**Affiliations:** 1 I-HAB, Institute of Human Virology Nigeria, Abuja, Nigeria; 2 University of Maryland School of Medicine, Institute of Human Virology, University of Maryland Baltimore, 725 West Lombard Street Suite, Baltimore, MD USA; 3 Integrated Biorepository of H3Africa Uganda, Kampala, Uganda; 4 Makerere University, Kampala, Uganda; 5 Clinical Laboratory Services, Wits Diagnostic Innovation Hub, University of the Witwatersrand, Johannesburg, South Africa; 6 National Human Genome Research Institute, National Institutes of Health, Bethesda, MD USA; 7 National Institute of Environmental Health Sciences, National Institutes of Health, Durham, NC USA; 8 Division of Immunology, Department of Pathology, Faculty of Health Sciences, University of Cape Town, National Health Laboratory Service, Johannesburg, South Africa

**Keywords:** H3Africa consortium, Biorepository, Capacity building

## Abstract

**Background:**

Biorepositories archive and distribute well-characterized biospecimens for research to support the development of medical diagnostics and therapeutics. Knowledge of biobanking and associated practices is incomplete in low- and middle-income countries where disease burden is disproportionately high. In 2011, the African Society of Human Genetics (AfSHG), the National Institutes of Health (NIH), and the Wellcome Trust founded the Human Heredity and Health in Africa (H3Africa) consortium to promote genomic research in Africa and established a network of three biorepositories regionally located in East, West, and Southern Africa to support biomedical research. This manuscript describes the processes established by H3Africa biorepositories to prepare research sites to collect high-quality biospecimens for deposit at H3Africa biorepositories.

**Methods:**

The biorepositories harmonized practices between the biorepositories and the research sites. The biorepositories developed guidelines to establish best practices and define biospecimen requirements; standard operating procedures (SOPs) for common processes such as biospecimen collection, processing, storage, transportation, and documentation as references; requirements for minimal associated datasets and formats; and a template material transfer agreements (MTA) to govern biospecimen exchange. The biorepositories also trained and mentored collection sites in relevant biobanking processes and procedures and verified biospecimen deposit processes. Throughout these procedures, the biorepositories followed ethical and legal requirements.

**Results:**

The 20 research projects deposited 107,982 biospecimens (76% DNA, 81,067), in accordance with the ethical and legal requirements and established best practices. The biorepositories developed and customized resources and human capacity building to support the projects. [The biorepositories developed 34 guidelines, SOPs, and documents; trained 176 clinicians and scientists in over 30 topics; sensitized ethical bodies; established MTAs and reviewed consent forms for all projects; attained import permits; and evaluated pilot exercises and provided feedback.

**Conclusions:**

Biobanking in low- and middle-income countries by local skilled staff is critical to advance biobanking and genomic research and requires human capacity and resources for global partnerships. Biorepositories can help build human capacity and resources to support biobanking by partnering with researchers. Partnerships can be structured and customized to incorporate document development, ethics, training, mentorship, and pilots to prepare sites to collect, process, store, and transport biospecimens of high quality for future research.

## Background

In 2010, the National Institutes of Health (NIH) and Wellcome Trust funded the Human Heredity and Health in Africa (H3Africa) consortium in partnership with the African Society of Human Genetics to study genetic and environmental determinants of disease and establish genetic infrastructure and training/capacity building for African scientists and institutions [1]. Independent African scientists and resource centers, who successfully competed for funding to support his/her genomic research or resource center became members of the consortium. Three biorepositories were among the resources supported by the consortium to house and distribute the subset of DNA biospecimen that were required of H3Africa scientists to deposit to promote and support future genomic research including Clinical Laboratory Services (CLS), the Integrated Biorepository of H3Africa Uganda (IBRH3AU), and the Institute of Human Virology H3Africa (I-HAB) located in Southern, Eastern, and Western Africa, respectively. These biorepositories have been in operation for over 10 years and have become a blueprint of biorepository operations, science, and management in Africa [2–6].

The hallmark of biorepositories is to receive, process, archive, and distribute well-characterized biospecimens and their annotated data to support the development of scientific research, medical diagnostics, and therapeutics [7]. The quality of archived biospecimens including their derivatives impacts the outcome of scientific research. Considering the high burden of diseases in Africa [8, 9], the role of biorepositories is crucial to the continent, especially in research and precision medicine given the importance of having quality biospecimens in the archives. Yet, despite advancements through international funding and partnerships, Africa is still undeveloped in biobanking and struggles with destitute laboratory systems, infrastructure, and logistics [4]. Considering the significance of depositing high-quality biospecimens to the biorepositories for successful downstream research applications [10], it was crucial for the three African regional biorepositories to provide the research/clinical sites with best practices oversight for sample management to achieve quality research outcomes. Sample management generally encompasses procedures for the collection, documentation, processing, and archiving of samples for future application. For the clinical and research centers saddled with such a chain of responsibilities to measure up, the biorepositories must assess to determine the strengths and weaknesses and provide resources to bridge gaps identified in preparation for biospecimen collection.

In this article, we describe the experiences of the H3Africa biorepositories in engaging research sites to prepare for the collection of high-quality biospecimens for the submission to H3Africa biorepositories. This effort represents a pivotal expansion in biobanking capacity development in Africa and will enable future novel research activities.

## Methods

**Fig. 1:**
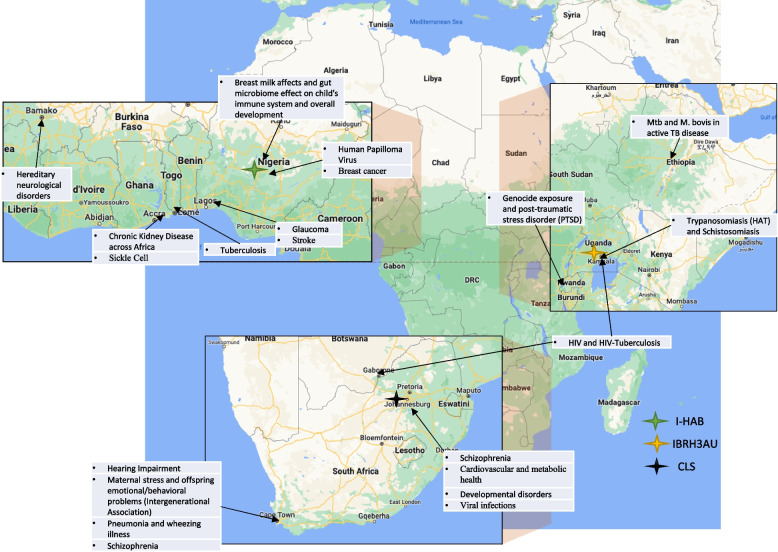
Research sites and assigned biorepositories

One objective of the H3Africa consortium was to provide high-quality DNA for future use to maximize the value of these hard-to-obtain specimens with high potential to provide information to the biomedical research community. Therefore, H3Africa required most H3Africa genomic research projects to obtain broad consent to deposit DNA for each study participant. Each of these projects was assigned to an H3Africa biorepository according to its study location (Fig. 1): CLS to projects within Southern Africa, IBRH3AU to projects within East Africa, and I-HAB to projects within West Africa. Researchers could also deposit other biospecimen types voluntarily for storage; however, the consortium does not require such biospecimens to be accessible for research. The biorepositories harmonized practices with research sites by needs assessment, harmonized and standardized documents and processes, trained and provided mentorship to bridge gaps, piloted all processes, and continuously monitored for improvement.

### Needs assessment

The biorepositories discussed biospecimen collection and deposit with assigned research sites, visited sites where feasible and reviewed relevant documents and processes to determine needs and devise strategies for support. The biorepository committee (The Committee), composed of NIH technical staff and the biorepositories’ PIs and staff, met bi-weekly during the pilot and early implementation phases and monthly thereafter to establish requirements and processes to accomplish biospecimen deposit and access. Each biorepository also met with the assigned research sites as needed to educate them in biobanking, services, and requirements and to discuss updates, challenges, remediation, and potential needs such as document development and training. Where feasible, H3Africa biorepositories visited research sites to become familiar with relevant processes and to conduct needs assessments. The biorepositories also reviewed relevant documents to ensure compliance and harmony among the various institutions and procedures, and to identify gaps. Document review varied according to need and included MTAs, consent forms, study protocols, standard operating procedures (SOPs), and supply lists. The biorepositories addressed gaps through document development, document revisions, training, and mentorship.

### Drafted documents for harmonization and standardization

The Committee harmonized documents to promote accountability, transparency, and consistency. The Committee assembled the Writing Subgroup to draft resource documents and the Data and Biospecimen Access Committee (DBAC) to develop requirements and processes for biospecimen access. The Writing Subgroup developed guidelines, SOPs, forms, minimal datasets, and other reference documents to harmonize and standardize processes. Similarly, the subgroup created a Material Transfer Agreement (MTA) as a resource for institutions that did not have MTAs or had limited experience.

### Training and mentorship

**Fig. 2:**
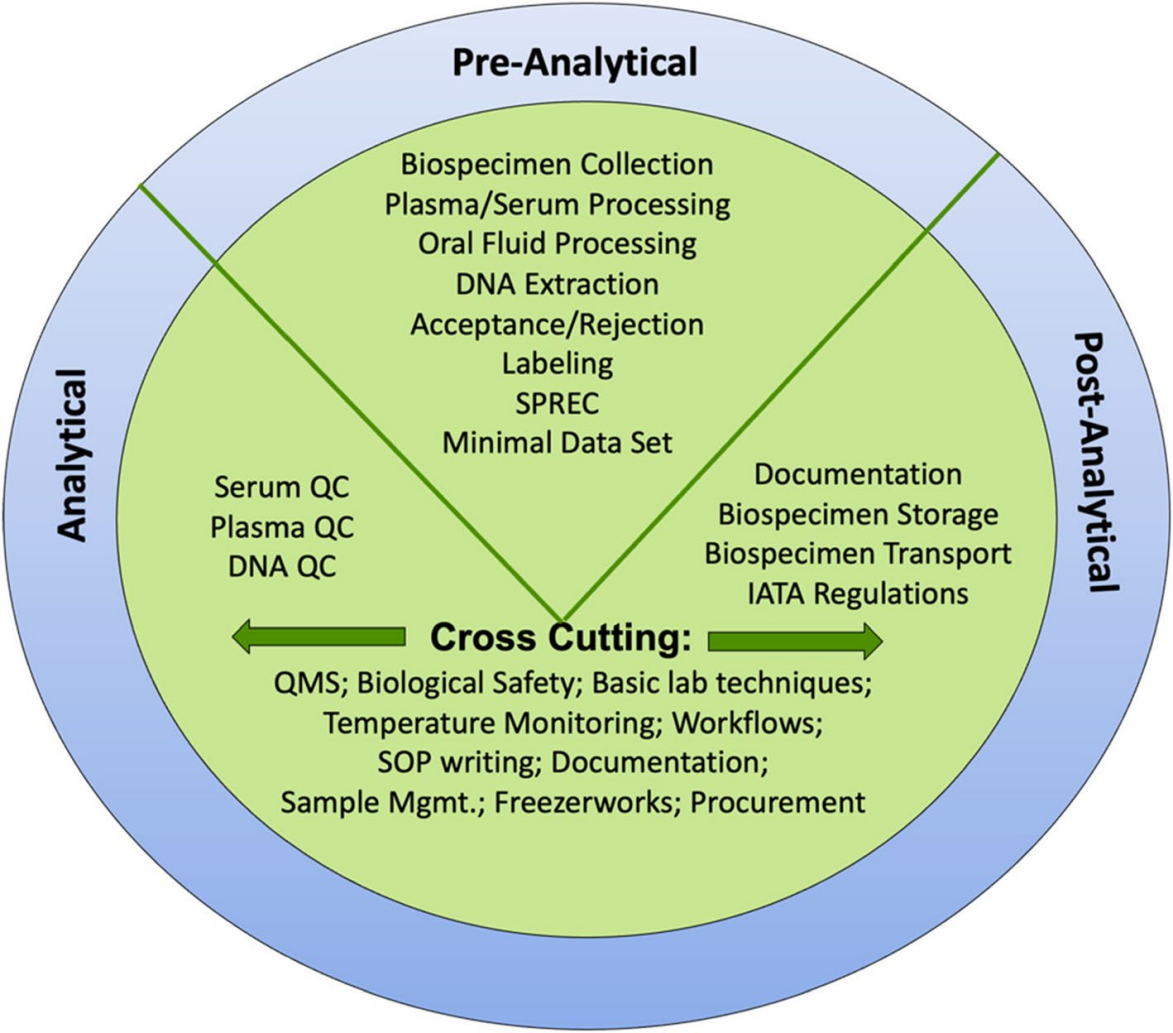
Training and mentorship topics

The H3Africa research staff involved in processes related to biospecimen deposits were trained in related pre-analytical, analytical, and post-analytical processes to ensure high-quality biospecimen and data. Training opportunities (Fig. 2) were aligned with project needs, as determined from meetings, on-site observations, document reviews, and principal investigator (PI) requests. Training aligned with International Society for Biological and Environmental Repositories (ISBER) best practices, established industry practices, and included theory, class exercises, practical exercises, and competency assessments. Biorepositories also mentored research staff in document development, workflow design, biological transport, laboratory procedures, and areas of improvement identified during training.

### Piloted ethical and legal procedures and requirements

#### Ethical requirements

H3Africa biorepositories established several mechanisms to ensure biospecimen use was consistent with approved project protocols, policies, procedures, and informed consent. H3Africa biorepositories sensitized their respective institutional review boards (IRB) through trainings, meetings, and/or site visits to promote biobanking awareness and advocate for policies and processes that intercalate biobanking needs and peculiarities: IBRH3AU-Uganda National Council of Science and Technology (UNCST) and Makerere University School of Biomedical Science Research Ethics Committee (SBS REC), I-HAB-National Health Research Ethics Committee (NHREC), and CLS-Human research ethics committee of the University of Witwatersrand. The biorepositories also requested protocols and informed consent to ensure compliance with ethical clearance.

H3Africa biorepositories developed documents and processes to protect the security and integrity of biospecimens and data. As mentioned, the MTA served as a resource that researchers could adapt and adopt to set limitations and expectations in material exchange. Likewise, the H3Africa Data and Biospecimen Access Guidelines provide biospecimen access requirements, processes, and criteria for selection and prioritization to ensure future researchers adhere to the ethical requirements.

#### Legal requirements

The biorepositories and researchers shared biospecimens and associated data according to universal legal requirements and legal requirements specific to the donor and recipient’s country and institution of origin. In adherence to the International Air and Transport Association (IATA) regulations, the Committee required shippers at research sites and biorepositories to attain and maintain IATA shipping certification and provided additional in-person training and mentorship as needed in IATA, biological packaging, and shipment. Shippers were certified in class 6.2 for biological, infectious substances and class 9 for dry ice as appropriate. The biorepositories investigated import/export requirements and obtained permits as required.

### Piloted biospecimen deposit

Although the biorepositories had piloted biospecimen deposits internally to test established procedures, documents, and logistics [11], the biorepositories also piloted processes required for biospecimen deposits with inexperienced researchers as needed. To prepare, the biorepositories developed a pilot protocol and a shipment checklist and reviewed them, the overall process, and the associated documents with the research sites. Biobanks and researchers met all the ethical and legal requirements prior to biospecimen or data exchange.

#### Research sites

**Fig. 3:**
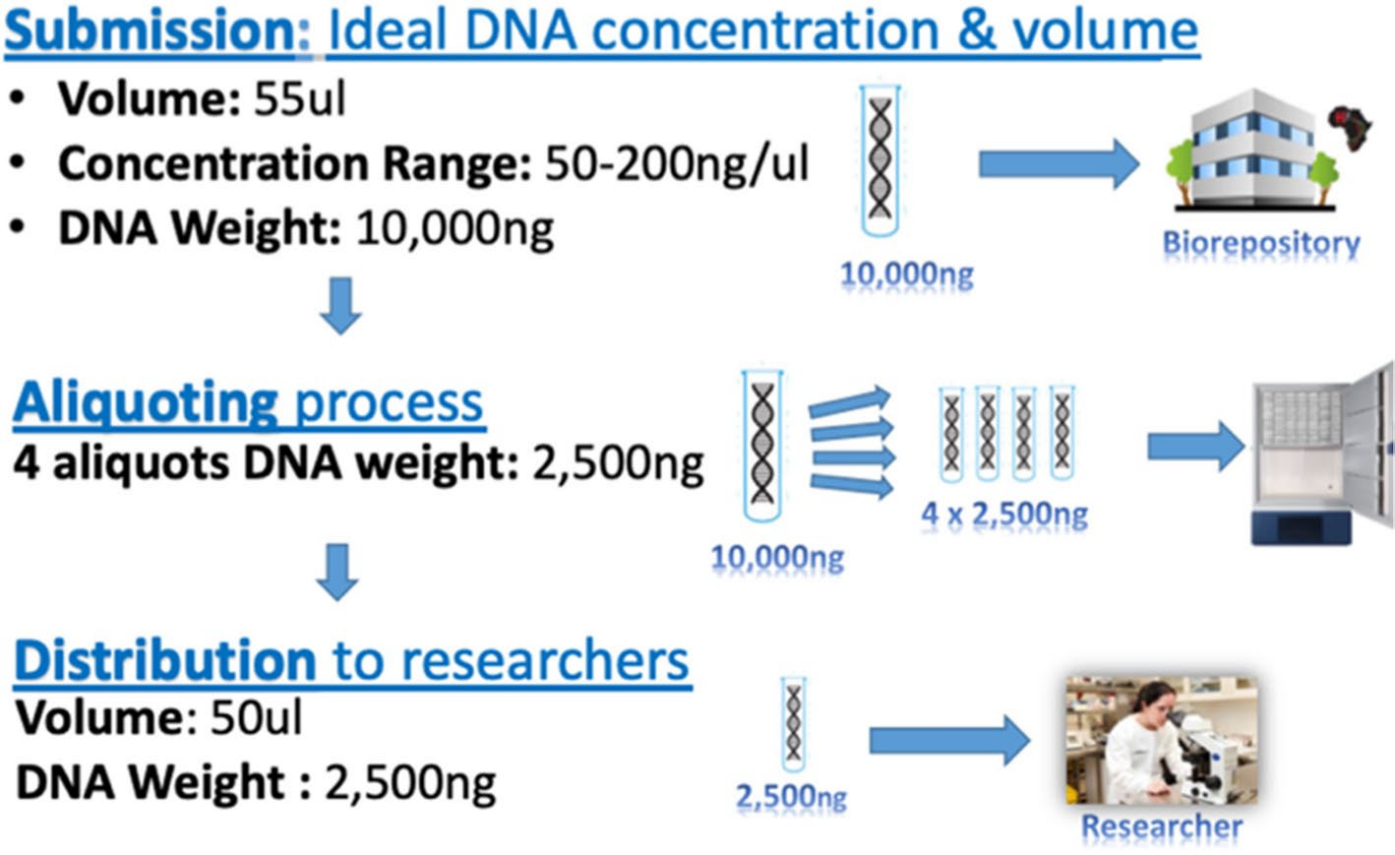
Biospecimen deposit guidelines—standardization from deposit to distribution

The research sites recruited participants, consented to them, collected biospecimens, processed, and temporarily stored participant biospecimens as part of their ongoing study protocol process. Research sites minimally included DNA as required, but some also included non-DNA biospecimens representative of the biospecimen types they would deposit during implementation. The Biospecimen Deposit Guidelines specified requirements for deposit such as labeling, quality control (QC), and minimum number of aliquots (Fig. 3).

For the pilot, the research sites prepared to deposit biospecimens to their assigned biorepositories. Table 1 summarizes the research sites’ locations, area of research, and biospecimen types deposited. Consult the H3Africa catalog, https://catalog.h3africa.org, for additional information about the research projects, such as study design. The research sites also completed and emailed the sample manifest and accompanying shipping documentation, scheduled biospecimen pick up, and emailed the expected date of shipment/pick up prior to shipment to the biorepository. The sample manifest included all the minimal dataset elements, as well as information relevant to the shipment such as aggregate data on sample types and associated shipping conditions. All biospecimens were shipped in accordance with IATA regulations. The sites shipped serum, plasma, and urine frozen (dry ice or Credo shipper-temperature controlled); whole blood at controlled ambient temperature; and DNA at a combination of controlled ambient, refrigerated, or frozen, in accordance with H3Africa standard operating protocols (SOPs). The sites used reusable quantitative temperature loggers (Sensitech, Beverly, MA, USA) and qualitative disposable 3TM Warmmark time–temperature indicators (Anaheim, TelaTemp, CA, USA) to monitor the shipping environment to determine if the cold chain was compromised (unless indicated otherwise).

**Table 1:** Research site locations, assigned biorepositories, and biospecimen types

Location	Brief description of research	Biospecimen types
IBRH3AU		
Uganda and Botswana	HIV and HIV-tuberculosis (TB)	DNA
Addis Ababa, Ethiopia	Active TB disease	DNA
Kigali, Rwanda	Genocide exposure and post-traumatic stress	DNA
Kampala, Uganda	Trypanosomiasis and schistosomiasis	DNA and plasma
I-HAB		
Cotonou, Benin Republic	Tuberculosis	DNA
Accra, Ghana	Chronic kidney disease	DNA, plasma, serum, buffy coat, red cells, oral fluids, and urine
Accra, Ghana	Sickle cell	DNA, plasma, serum, buffy coat, red cells, and urine
Bamako, Mali	Hereditary neurological disorders	DNA
Abuja, Nigeria	Human papillomavirus	DNA
Abuja, Nigeria	Breast cancer	DNA
Ibadan, Nigeria	Glaucoma	DNA
Ibadan, Nigeria	Stroke	DNA, serum, plasma
Plateau, Nigeria	Breast milk affects and gut microbiome	DNA
CLS		
Cape Town, SA	Hearing impairment	DNA
Cape Town, SA	Maternal stress and offspring emotional and behavioral problems	DNA
Cape Town, SA	Pneumonia and wheezing illness	DNA
Cape Town, SA	Schizophrenia	DNA
Johannesburg, SA	Cardiovascular and metabolic health	DNA
Johannesburg, SA	Developmental disorders	DNA
Johannesburg, SA	Viral infections	DNA

#### Biorepositories

Once biorepositories received the email alert of the planned biospecimen shipment, the staff prepared for biospecimen deposit. The biorepository reviewed the documents for completeness and accuracy and contacted the researcher if needed for clarification or modification. Upon shipment, the biorepository tracked the shipment daily to monitor the expected date of arrival (DOA) and to detect and rectify potential arising issues such as delays in customs. The biorepository also tracked the Sensitech temperature loggers daily to ensure that the cold chain was not compromised and to intervene should issues arise. Upon receipt of the shipment, the biorepository observed the temperature or indicator of the temperature monitor, subjected the biospecimen to acceptance/rejection criteria, and compared them to the accompanying manifest. Next, the biorepository completed the Shipment Receipt Confirmation and Query Form, documenting any non-conformities, and forwarded it to the researcher to confirm receipt and communicate and resolve non-conformities.

After completing the biospecimen receipt process, the biorepository performed QC for 10% of shipped biospecimen and sub-aliquoted DNA according to the Biospecimen Deposit Guidelines and SOPs and stored the biospecimen. For *quality control purposes*, the biorepositories determined DNA concentration at an absorbance of 260 nm, determined purity using the 260/280 absorbance ratio, and conducted agarose gel electrophoresis to determine the integrity of the samples [12, 13]. Biorepositories used visual grading for plasma and serum to determine hemolysis and turbidity, and pH and turbidity for urine. Each biorepository used a *laboratory information management system (LIMS)* to collect and store biospecimen-associated data, importing data from the sample manifest and adding data for sample location, in-house QC, aliquoting, etc. The LIMS included Freezerworks (Dataworks Development, Seattle, WA) for I-HAB and IBRH3AU, and LDMS (Frontier Science Foundation, Amherst, NY) for CLS.

### Continuous Quality Improvement (CQI)

The biorepositories and research sites partnered to ensure the gains accomplished through the pilot and remedial actions were maintained through program implementation. Both teams reviewed data for accuracy and completion before and following shipment receipt and communicated discrepancies within 1 week for resolution. The teams also tracked shipments and monitored environmental conditions minimally once per day until delivery. In line with the Biospecimen Deposit Guidelines, the biorepositories performed QC on 10% of the DNA received, if < 90% of those results were beyond A260/280 of 1.7–2.0, or of high molecular weight by gel electrophoresis, the biorepository tested the remaining 90%. For subsequent shipments, 100% QC was conducted until ≥ 90% of the QC results were of acceptable quality. The biorepository also informed the researcher of the discrepancies, shared its in-house results, assisted with root-cause analysis, and supported corrective and preventive actions. The biorepositories continue to meet with the research staff to discuss the challenges and strategies for improvement.

## Results

The three H3Africa biorepositories engaged with their 20 respective research sites to build capacity and effective and efficient processes towards the generation and sustainability of high-quality, well-annotated biospecimens. Through needs assessment, training, mentoring, piloting, and continuous improvement, the biorepositories and researchers achieved a deposit of 107,982 biospecimens, including 81,067 DNA and 26,915 non-DNA biospecimens, through April 1, 2023. The biospecimen had 91.5% acceptable quality.

- *Developed 34 guidelines and SOPs* which cover ethical and legal considerations to sample collection, processing, and shipment (Table 2) to standardize processes.
- *Trained 176 research staff* in over 30 topics related to biobanking (Table 3). I-HAB trained and mentored 72 staff in over 30 topics including sample collection, processing, shipment, and IATA shipping guidelines. IBRH3AU trained 45 staff in sample collection, processing, and shipment. CLS trained 59 staff in GLP and local shipment.
- *Sensitized ethical bodies*, contributing to ethical advancements: NHREC instituted registration requirements, monitoring site visits, and incorporated associated ethical reviews for biobanks to operate in Nigeria. I-HAB was the first biorepository to undergo these processes. The UNCST and SBS REC to which IBRH3AU subscribes set up guidelines and ethical policies under which IBRH3AU operates. IBRH3AU’s ethical approval is subjected to annual review and renewal in addition to site monitoring visits that ensures compliance to the required standard. In South Africa, CLS was the first biorepository to undertake accreditation via the ethics body; thus, the checklist and process for accreditation was pioneered using CLS.
- *Established MTAs* and reviewed ethical documents for all 20 H3Africa projects, including projects with more than one research site.
- *Attained three (3) import permits* for IBRH3AU; however, I-HAB and CLS did not require import permits.
- *Conducted and evaluated 6 shipping pilot exercises* (deposited 4052 biospecimens) and *3 virtual pilot exercises* and provided feedback.
- *Implemented CQI to address issues* identified through routine monitoring.
- *Biospecimen deposit by site*: CLS 26,938, IBRH3AU 22,142 (96.8% DNA), and I-HAB 58,902 (55.5% DNA) (Table 3).

**Table 2:** Documents developed by the H3Africa Biorepositories

Guidelines	Forms
Phlebotomy	Shipment notification and manifest
Biospecimen storage	Shipment query notification
Biospecimen shipping and transportation	
Biospecimen deposit	
Biospecimen and data access	
Sample collection	Sample processing
Phlebotomy	Plasma and serum processing
PAXgene—DNA and RNA	PAXgene DNA and RNA isolation
Oragene saliva—DNA and RNA	Oragene saliva—DNA and RNA isolation
Tempus blood RNA	Tempus blood RNA isolation
PBMC	PBMC isolation
Urine collection and processing	DNA and RNA extraction from blood derivatives
	Destruction of human biospecimens
Sample quality control	Biospecimen transport
Acceptance and rejection criteria	Biospecimen submission
Nucleic acid quality assessment	Identification, labeling, and tracking
QC for plasma and serum	Biospecimen shipping and transportation
QC validation for plasma and serum	Material transfer agreement

**Table 3:** Outcomes of research site engagement

Biorepository	H3Africa projects	Persons trained	Import/export permit obtained	MTA	Pilots	Pilot deposit	Total DNA/non-DNA deposit
I-HAB	9	72	0/0	10	6	4052	32,687/26,215
IBRH3AU	4	45	3/0	6	0	–	21,442/700
CLS	7	59	0/0	–	3	–	26,938/0

These documents address future data and biospecimen modifications, derivations, distribution, roles and responsibilities, and related issues. The guidelines reflected best practices and defined biospecimen requirements, and SOPs covered common processes such as biospecimen collection, processing, quality control (QC), storage, and transportation. Documents are accessible from the H3Africa Consortium Documents website (https://h3africa.org/index.php/consortium/consortium-documents/) and the resources page of the H3Afica biorepository website (www.h3africa.org).

CQI helped to identify areas of improvement and to develop and implement remediation strategies.

- CLS upscaled staff skills in DNA extraction and quality control processes. Trainings in shipping ensured that sample integrity was maintained during the shipment process.
- IBRH3AU helped improve processes at research sites by providing recommendations for samples received, e.g., packaging condition, concentration, and purity.
- I-HAB helped improve the quality of DNA from the initial pilot to subsequent shipments through training, mentorship to newly hired staff, and providing specifications for centrifuges and biospecimen collection and storage supplies. DNA concentration improved from 15–69.66 ng/μl to 78.72 to 514 ng/μl, and purity improved from 38% acceptability to 90%.

## Discussion

The 10 years (2013–2023) of research site engagement has demonstrated that biorepositories can build the capacity of research sites in Africa, to prepare high-quality, well-annotated biospecimen through needs assessment, standardization and harmonization, training, mentorship, pilot, and CQI. Methods of information gathering such as meetings, visits, and document review are useful to determine and communicate goals, requirements, strengths, and weaknesses and to target training and mentorship accordingly. Through consortium meetings, WG meetings, site engagement meetings, on-site visits, and document review, the biorepositories recognized the great diversity among research sites regarding laboratory and biobanking practices. To meet the performance standards set for biorepositories [10, 14], addressing operational diversities that fall below the best practices is essential. For example, several of I-HAB’s assigned sites were inexperienced in laboratory-related procedures, whereas several of CLS’s assigned sites were experienced. The disparity implies that the techniques for engagement must be sensitive enough to detect differences in site needs and flexible enough to address them. Here, the I-HAB sites received training and mentorship in all procedures from safety, QMS, and documentation through specimen collection through deposit, whereas the CLS site received onsite training in good laboratory practices (GLP), local shipment, and corresponding documentation.

The biorepositories achieved process standardization and harmonization, through clear documentation and communication. The biorepositories used standard meetings to develop guidelines to promote consistency in biospecimen deposit and access processes and to communicate those requirements to the consortium and research sites. The biorepositories used information gathered from research sites, such as types of biospecimen to be collected and processed, to determine the topics for reference documents. This supports the findings of other biorepositories [15–17] that reported successes after the implementation of quality operational measures. The biorepositories furthered streamlined processes and built efficiency by establishing a minimal dataset and format and biospecimen manifest for consistency in data.

Training and mentorship are critical to bridge the gaps in ethical, legal, and biobanking-related requirements, processes, and procedures. Although guidelines and SOPs are useful for building capacity, training, particularly didactic training comprising theory and practical exercises, was required to exemplify what was written in technical SOPs. For example, some sites who attended online IATA training and read shipping SOPs for IATA and biological packaging requested on-site training and mentorship for their first shipment. Furthermore, technical procedures may also require additional re-training and/or mentorship to account for slow implementation and/or staff turnover, such was the case where one of I-HAB’s research sites deposited poor-quality DNA after the trained staff left; a rapid intervention from biorepository staff helped drastically improve quality.

Pilots are useful to test workflows, processes, and procedures to identify areas of improvement before implementation [11], especially when any of these or the implementers are new. By piloting all the processes from ethical and legal requirements through biospecimen deposit and analysis, the team learned that ethical approvals and MTA agreements can take months to establish, especially in institutions that are unfamiliar with concepts of biospecimen, data, and benefits sharing required for biobanking, and collaborative research. Also, the team would not have identified and addressed issues with DNA biospecimen quality. Above all, pilots provide the opportunity to test all the moving parts to an overall process and move from theory to feasibility.

CQI is a repetitive process of monitoring and evaluation that should be done throughout the entire project. Observations from meetings, site visits, training, mentorship, and pilot were analyzed to identify and rectify areas requiring improvement. CQI is the cornerstone towards sustainable deposit of high-quality, well-annotated biospecimen and data.

The biorepositories witnessed several challenges during the implementation of the biorepository program. Initially, H3Africa researchers were unfamiliar with the knowledge of biobanking and its benefits. This led to some of them declining biospecimen deposits as against the funding requirement. However, through advocacy and training, all the research sites successfully deposited DNA in the biorepositories, including non-DNA biospecimens, and used other biorepository services like DNA extraction, document development, and training. In addition, the biorepositories also experienced delays establishing MTAs for some sites as some institutions had never dealt with concepts of MTAs or biospecimen, data, or benefit sharing. The biorepositories resolved this by preparing a template MTA as a resource and applying for MTAs early in site engagement and before the initiation of the shipping or virtual pilots. The COVID-19 pandemic also delayed sample deposit activities due to shipping embargos, shutdowns, disruption to manufacturing and supply chains, and limited flights even as shipping resumed. The biorepositories, research projects, and NIH eventually developed plans which led to the funding extension to achieve complete biospecimen deposition before the program ended. Meanwhile, this paper is limited to the experience of the three biorepositories and their assigned research sites within the consortium. The outcomes might be different in other circumstances; thus, it is imperative to assess prior to developing a plan of engagement.

## Conclusions

The H3Africa regional biorepositories successfully developed capacity of the partner research sites through document development, standardization, training, piloting, and establishing legal and ethical practices to produce high-quality samples of African origin for archival and distribution across Africa and the world. Knowledge of biobanking and its related activities is limited in low- and middle-income countries; however, biorepositories can help build human capacity and resources to support biobanking by partnering with researchers. Partnerships can be structured and customized to incorporate document development, ethics, training, mentorship, and pilots to prepare sites to collect, process, store, and transport biospecimens of high quality for future research. As a result, the H3Africa biorepositories have over 80,000 high-quality DNA aliquots and associated genomic data available from individuals across 10 countries consented responsibly by submitting requests through the catalog (https://catalog.h3africa.org) which is controlled by the Data and Biospecimen Access Committee via documented procedures and criteria [18].

H3Africa funding ended in 2023; however, the biorepositories continue to meet, collaborate, and share knowledge, resources, and opportunities, while continuing to operate as independent, fully operational biorepositories. Sustainability is at the core of each biorepository’s structure, through a blended funding model which consists of fee-for-service, academic/institutional funding, and grant funding. The biorepositories all support government and non-H3Africa projects, participating in initiatives as diverse as surveillance, trials, and research—including prospective studies. The biorepositories also conduct community outreach, media campaigns, and participate in global, regional, national, and local organizations and meetings to advocate, educate, and promote biobanking services in Africa and beyond.

## Data Availability

Not applicable.

## Abbreviations

AfSHG: African Society of Human Genetics
CLS: Clinical Laboratory Services
COVID-19: Coronavirus disease 2019
CQI: Continuous quality improvement
DOA: Date of arrival
DBAC: Data and Biospecimen Access Committee
DNA: Deoxyribonucleic acid
GLP: Good Laboratory Practices
H3Africa: Human Heredity and Health in Africa
HIV: Human immunodeficiency virus
IATA: International Air Transport Association
IBRH3AU: Integrated Biorepository of H3Africa Uganda
I-HAB: Institute of Human Virology Nigeria
IRB: Institutional Review Board
ISBER: International Society for Biological and Environmental Repositories
LIMS: Laboratory Information Management System
MTA: Material transfer agreements
NIH: National Institutes of Health
NHREC: National Health Research Ethics Committee
PI: Principal investigator
QC: Quality control
RNA: Ribonucleic acid
SA: South Africa
SBS REC: Makerere University School of Biomedical Science Research Ethics Committee
SOP: Standard operating procedure
SPREC: Standard Preanalytical Coding for Biospecimen
TB: Tuberculosis
UNCST: Uganda National Council of Science and Technology
